# Rupture of the meniscofibular ligament

**DOI:** 10.1186/1749-799X-5-35

**Published:** 2010-05-18

**Authors:** Koray Unay, Korhan Ozkan, Irfan Esenkaya, Oguz Poyanli, Kaya Akan

**Affiliations:** 1 Department of Orthopaedic and Traumatology, Goztepe Research and Training Hospital, Istanbul, Turkey

## Abstract

The meniscofibular ligament is an anatomically defined ligament of the knee in humans. However, there are no data regarding the prognosis following injury to this ligament. Our case was a 42-year-old man who presented at our clinic with pain of the lateral side of his left knee. MRI of his left knee revealed the rupture of the meniscofibular ligament. The mechanism of injury was consistent with anatomical and mechanical studies of the meniscofibular ligament. The patient was treated conservatively for 1 year, but his pain did not resolve completely. A case series of patients with the same injury is required to establish an effective treatment for this rare injury.

## Introduction

The lateral collateral ligament of the knee consists of superficial, intermediate, and deep layers, which contain defined ligaments and pericapsular tissues. The meniscofibular ligament is an anatomically defined ligament of the knee in humans [1-3]. However, there are no data regarding injury to this ligament and the prognosis. Bozkurt et al. confirmed the presence of this ligament in all the subjects examined in their study, demonstrating increased tension of this ligament during dorsiflexion of the ankle and describing its protective function against varus forces to the knee and external rotation forces to the leg [1]. Furthermore, Bozkurt et al. emphasized the need for biomechanical, radiological, and clinical studies of the meniscofibular ligament [1].

Although anatomical and mechanical characteristics of the meniscofibular ligament have been described, to the best of our knowledge, no case of injury to this ligament has been reported in the literature. Here, we describe a case of meniscofibular ligament injury and present our preferred treatment method and its outcome.

## Case Presentation

**Figure 1:**
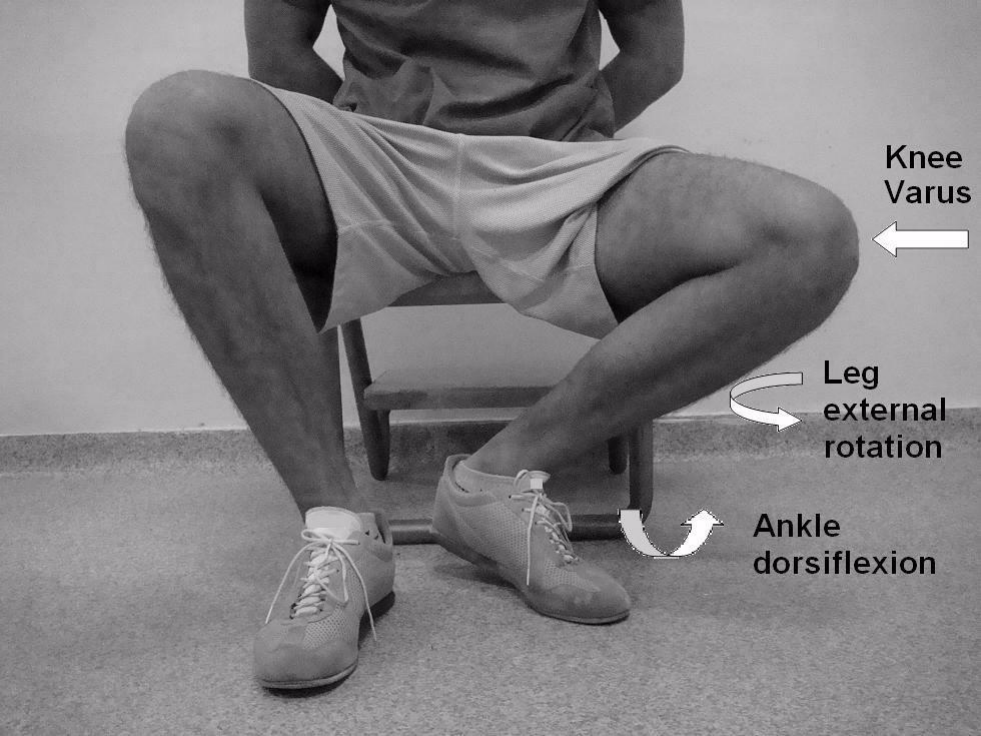
**Sitting position of the patient in the car**.

A 42-year-old male taxi-driver presented to our clinic with pain of the lateral side of his left knee. He had no history of trauma and did not report any rheumatological disease or previous knee surgery in his medical history. He stated that the pain was usually more intense when he waited in the car for a customer. His position in the car seat while waiting for a customer is depicted in Figure 1. This position forced his ankle into dorsiflexion, his knee into varus, and his tibia into external rotation.

**Figure 2:**
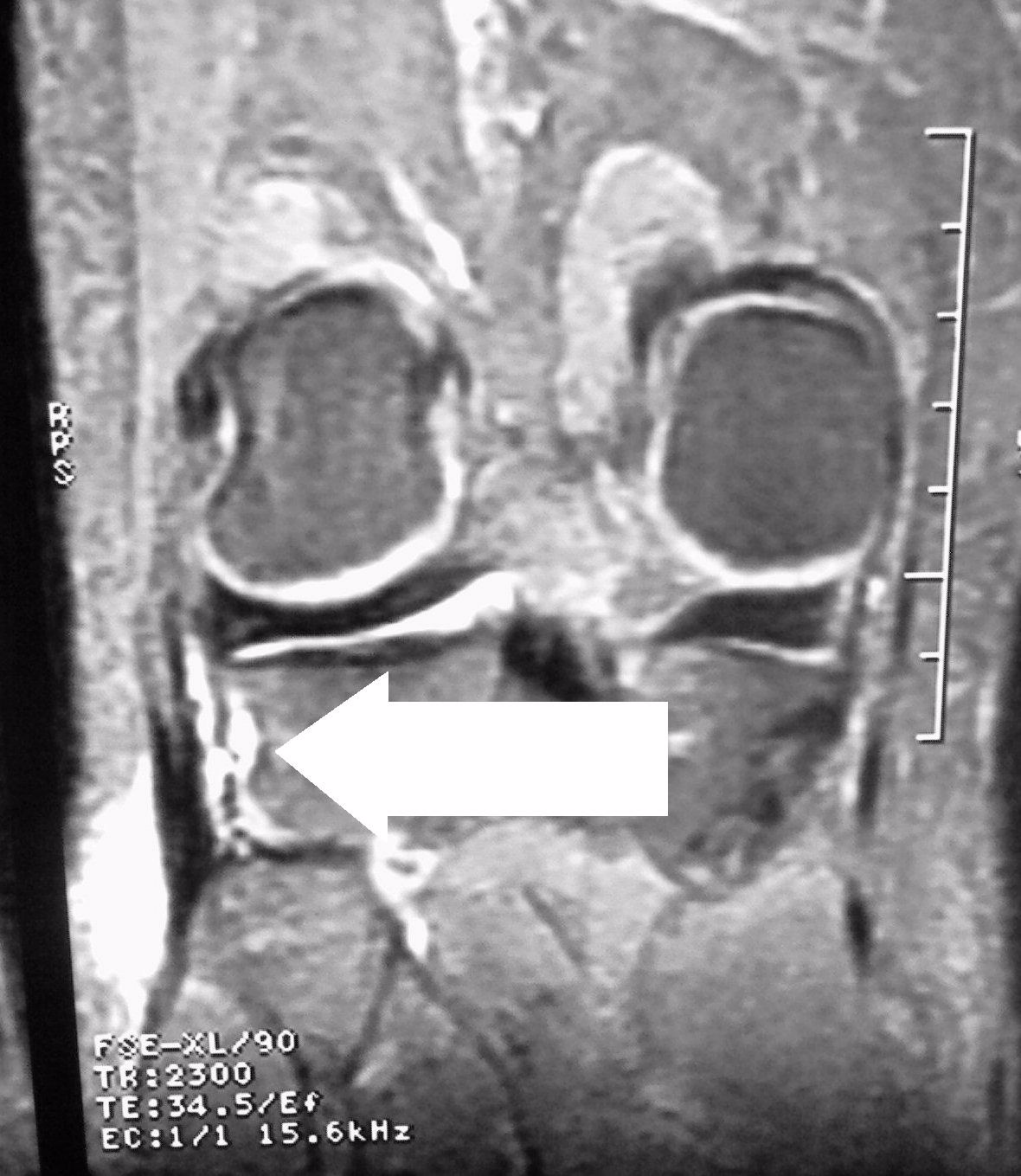
**Coronal section of MRI of the left knee**. Arrow: ruptured meniscofibular ligament.

**Figure 3:**
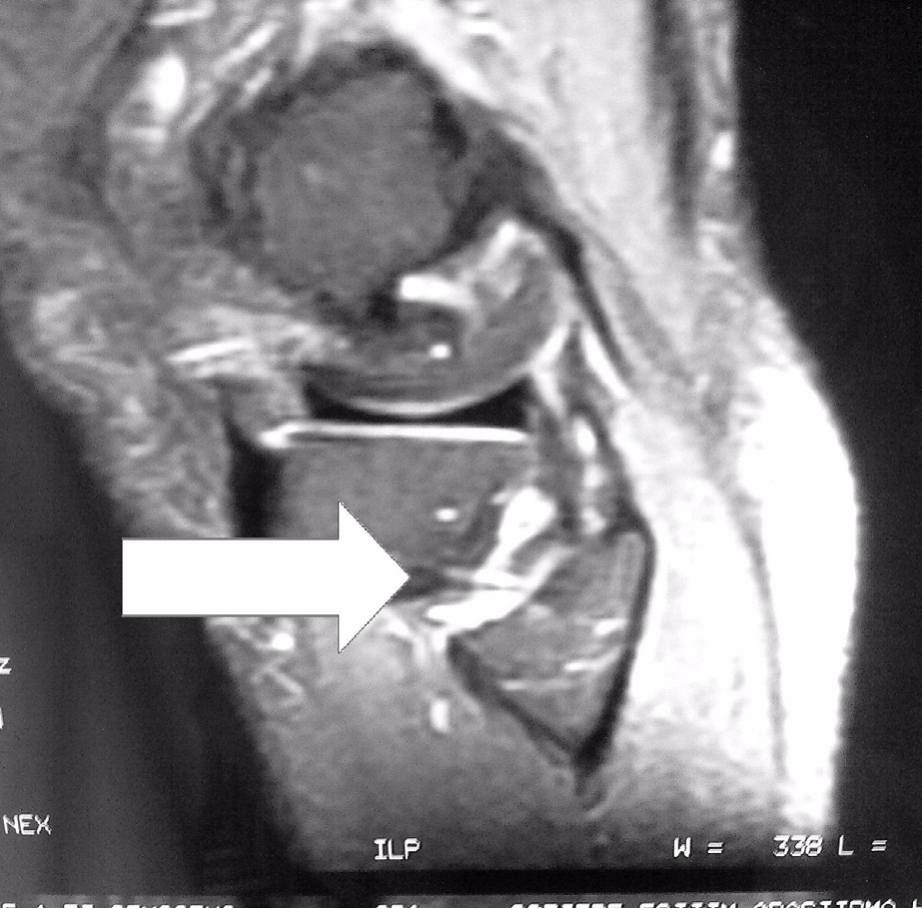
**Sagittal section of MRI of the left knee**. Arrow: ruptured meniscofibular ligament.

Tenderness at the head of the fibula and at the periphery of the lateral meniscus during palpation was the only positive finding during physical examination of the left knee. Tests for postero-lateral knee instability were negative. There was no pain or limp during walking. Pain occurred only during squatting and kneeling. Lower extremity neurological examination was intact. Peroneal nerve entrapment and radiculopathy-discopathy was not found. Antero-posterior radiography of his left knee showed no abnormalities. MRI of the knee (Figure 2 &3) revealed the rupture of the meniscofibular ligament. No other pathology was detected by MRI. The inclination of the proximal fibular joint was measured as 35 degrees; as the angle was greater than 20 degrees, it was classified as an oblique proximal fibula [3].

Bed rest was recommended for the first 10 days, with no weight-bearing and the use of two crutches. The knee was stabilized with a knee brace for 6 weeks, and a non-steroidal anti-inflammatory drug, diclofenac sodium 1 × 75 mg/day, was used for 3 weeks. The patient was allowed to return to his job after 3 weeks. He was advised to not sit for a long time in the above-mentioned position in the car seat. His pain gradually declined but persisted for 4 months. He was followed up for 1 year. At his last visit, the pain was considerably reduced because he had not sat in the above-mentioned position, but the pain had not subsided completely.

## Discussion

In an anatomical study, Bozkurt et al. described the position at which the meniscofibular ligament is in tension [1]. According to its anatomical structure, the proximal fibula is classified as oblique when the inclination of the joint is greater than 20 degrees or horizontal when the inclination of the joint is less than 20 degrees [3]. A horizontal proximal fibula allows a wider range of rotation [1,3]. Bozkurt et al. demonstrated that the meniscofibular ligament is thicker in subjects with a horizontal proximal fibula and rather slender in those with an oblique proximal fibula. Dorsiflexion of the ankle has been shown to create tension in the meniscofibular ligament. A protective function against varus and external rotation forces to the knee has been described for the meniscofibular ligament [1]. The sitting position described by the patient exactly matches the position that creates meniscofibular ligament tension (Figure 1). We believe that repetitive minimal stretching was responsible for the ligament rupture.

The mechanical significance of the ligament may be limited to its support of the lateral group of ligaments, but the meniscofibular ligament may also have a role in posterolateral corner and lateral meniscus injuries. Although the pain in the present case was considerably reduced after treatment, it had not subsided completely at the end of 1 year. This implies that the rupture of this ligament does not have a simple clinical prognosis.

We have presented the treatment and clinical prognosis of a patient with rupture of the meniscofibular ligament. This is the first time that this injury is reported. The mechanism of injury in our patient was consistent with previous mechanical and anatomical studies of this ligament. This case was the first clinical description suggesting a mechanical role for the meniscofibular ligament. Pain was considerably reduced after 1 year of conservative treatment but had not completely resolved. A case series of patients with the same injury is required to define the prognosis of this injury.

## List of abbreviations

MRI: Magnetic resonance imaging.

## Competing interests

The authors declare that they have no competing interests.

## Authors' contributions

KU conceived the idea and co-wrote the paper; KO and IE writing and aided in the editing of the manuscript; OP colleted the references and analyzed them and KA design the manuscript and edited the manuscript. All authors read and approved the final manuscript.

## Consent

Written informed consent was obtained from the patient for publication of this case report and accompanying images. A copy of the written consent is available for review by the Editor-in-Chief of this journal.
